# Intrahepatic cholangiocarcinoma trends and treatment lines: real-world evidence from the French National Hospital Discharge database

**DOI:** 10.1016/j.esmogo.2025.100152

**Published:** 2025-03-04

**Authors:** M. Delaye, B. Grenier, A. Lièvre, C. Neuzillet

**Affiliations:** 1 Department of Medical Oncology, Gastrointestinal Oncology, Institut Curie, Université Versailles Saint-Quentin-Université Paris-Saclay, Saint-Cloud, France; 2 Association pour l'étude des Cancers et Affections des voies Biliaires (ACABi), Paris, France; 3 Epidemiology Department, Heva, Lyon, France; 4 Department of Gastroenterology, University Hospital Pontchaillou, Rennes 1 University, INSERM U1242 “Chemistry Oncogenesis Stress Signalling”, Rennes, France

**Keywords:** biliary tract cancer, real-world data, CisGem, FOLFOX, artificial intelligence

## Abstract

**Background:**

Little is known about the therapeutic trajectory of patients treated in hospitals for intrahepatic cholangiocarcinoma (iCCA) and patterns of care in daily clinical practice.

**Patients and methods:**

An observational retrospective study was conducted on the French National Hospital Discharge Database. All patients with a new diagnosis of iCCA who had a first hospital stay (S1) from January 2016 to December 2021 were included. They were followed up until December 2021, or in-hospital death, whichever occurred first. Crude annual hospitalization rates were computed. Treatment lines were identified with an artificial intelligence algorithm [Analysis of Treatment Lines using Alignment of Sequences (ATLAS)]. A multistate model was used to compute the transition rates between lines.

**Results:**

Overall, 13 491 patients were included and the mean (standard deviation) follow-up duration was 13.1 months (17.9 months). The median age at S1 was 72.0 years and 55.9% were male. Nearly 20.7% were admitted via emergency services for S1, and 32.1% had metastases. Between 2016 and 2021, the crude annual rate of new iCCA cases increased from 3.08 [95% confidence interval (CI) 2.94-3.24] in 2016 (*n* = 1598) to 4.12 (95% CI 3.95-4.29) per 100 000 adult person-years in 2021 (*n* = 2188). Among 4855 patients receiving first-line systemic therapy (L1), 37.7% (95% CI 36.0% to 39.3%) received a second-line 2 (L2) during the follow-up. The median time between the start of L1 and the beginning of L2 was 7.0 months.

**Conclusions:**

This study provides up-to-date national real-world data on iCCA, revealing an increasing burden year by year in France, a poor outcome of patients with iCCA on L1 systemic therapy, and the low proportion of patients receiving an L2.

## Highlights

• The incidence of iCCA is increasing in France.
• Our real-world study confirms the poor outcomes and the low percentage of patients with iCCA receiving second-line treatment.
• Artificial intelligence improves the information obtained from the Hospital Discharge Database.

## Introduction

Intrahepatic cholangiocarcinoma (iCCA) accounts for 10%-30% of biliary tract cancers (BTCs) and represents 10% of primary liver cancers.^1^ Several recent observations have reported an increasing incidence in Western countries while other BTCs (extrahepatic cholangiocarcinoma, gallbladder carcinoma) seem to be stable.^2^, ^3^, ^4^ In case of locally advanced and unresectable or metastatic disease, the standard of care is mainly based on chemotherapy [CisGem: cisplatin and gemcitabine in the first-line setting (L1) and FOLFOX: fluorouracil and oxaliplatin in the second-line setting (L2)]. However, in recent years, immunotherapy and targeted therapies have been developed for BTC.^4^, ^5^, ^6^, ^7^, ^8^, ^9^ The reference treatment in L1 is now the combination of CisGem with an immune checkpoint inhibitor (durvalumab or pembrolizumab), and targeted therapies are preferred over FOLFOX in L2 in case of identified druggable alterations (IDH1, FGFR2, HER2, BRAF, and NTRK). These therapies have been approved based on clinical trials conducted in selected patients who are unlikely to be representative of the real-world population of patients with BTCs in terms of age, comorbidities, and disease complications. Real-world data are therefore critical to provide additional documentation of patient outcomes and treatment efficacy and tolerance.

Real-world data can be generated by analyzing data from retrospective or prospective cohorts, but are often limited by a small number of patients, and the results are highly dependent on the population included, which in turn depends on the hospitals participating in the cohort; in addition, patients receiving only supportive care are often excluded or underrepresented.^10^^,^^11^ Data can also be generated from national databases that include all patients with a specific pathology across the entire country. However, the extent and accuracy of the data available depends on the country and the design of the database. In 2022, our group published data from the French nationwide hospital discharge database [Programme de Médicalisation des Systèmes d’information (PMSI)] that included all patients with a new diagnosis of iCCA in France between 2014 and 2015 with a 2-year follow-up.^3^ These data from 3650 patients with iCCA allowed us to better understand the trajectories of iCCA patients, but the therapeutic strategy has changed since then (e.g. validation of FOLFOX as standard L2 and capecitabine in the adjuvant setting), and the analytical tools used did not allow us to study the type and sequence of chemotherapy received by the patients.

The aim of our study was to generate updated real-world data of patients with a new diagnosis of iCCA using the French nationwide hospitalization database and to use IA-based tools to better analyze the treatment sequences received by patients.

## Patients and methods

### Data source

Data were extracted from the nationwide hospitalization database (PMSI) which comprehensively and exhaustively records all hospitalizations in France in all types of care facilities for reimbursement purposes.^12^^,^^13^ Each patient included was identified by a unique identification number of anonymization, and data on her/his age, gender, type of care facility, diagnoses [coded according to the World Health Organization’s International Classification of Diseases, 10th revision (ICD-10)], medical and surgical interventions, duration of hospitalization stay, and vital status (alive or dead) were available.

### Study design

All patients with a new diagnosis of iCCA who had a first hospital stay (S1, index date) from 1 January 2016 to 31 December 2021 were included. They were followed up until the end of the study period (31 December 2021), or in-hospital death, whichever occurred first. Patients were included if aged ≥18 years and had been hospitalized (including day hospital) with a C221 code (intrahepatic bile duct carcinoma) as the main or related diagnosis during the period of inclusion. The main exclusion criteria were the presence of one of the following ICD-10 codes: C220 (hepatocellular carcinoma), C227 (other specified liver carcinomas), C18 (malignant tumor of the colon), C25 (pancreatic malignancy), C21 (malignant tumor of the anus and anal canal), and the presence of cholecystectomy or biliary drainage during follow-up to eliminate alternative primary tumor/metastasis from extrahepatic primary, or perihilar and extrahepatic cholangiocarcinoma that may have been miscoded.

The primary endpoint was the number of new iCCA cases during the period of interest. The secondary endpoints were demographic characteristics of the patients, their comorbidities, disease characteristics during S1, disease complications, cumulative rate of metastases in patients that had been operated, time to next treatment (TTNT, defined as the time between the initiation of line *N* and the initiation of line *N* + 1), and overall survival (OS).

### Statistical analyses

Quantitative variables were described using the number of patients (without missing data), mean with standard deviation, and median with minimum and maximum or interquartile range (IQR). Categorical variables were described using the percentage of patients by category. All confidence intervals (CIs) were two-sided and 95% (95% CI). CIs for prevalence and incidence were calculated using the normal approximation or exact Poisson distribution, as appropriate. As all treatments were recorded in the database, no missing data were replaced. Analyses were carried out using SAS Software, version 9.4 (SAS Institute Inc., Cary, NC, USA) or Python (version 3.7.3; R Foundation, Vienna, Austria).

**Figure 1:**
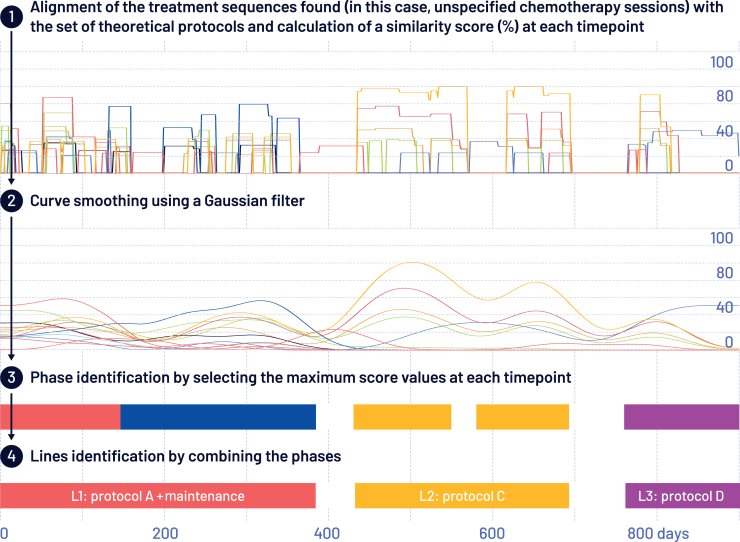
Overview of the methods used for the analysis.

To calculate the cumulative recurrence rate in patients that had been operated on, a cumulative incidence function was carried out to evaluate the first occurrence of metastasis or chemotherapy as an event of interest in patients operated on and not presenting with metastasis codes at S1, as well as over the pre-date index period.^14^ An estimate based on the Nelson–Aalen method, taking into account the competing risk of death on the occurrence of metastases, was implemented. We used the previously published Analysis of Treatment Lines using Alignment of Sequences (ATLAS) algorithm, designed by HEVA, to identify the treatment lines of patients with iCCA^15^^,^^16^ (Figure 1). In brief, all protocols/guidelines/management recommendations were listed by the scientific committee (MD, CN, or AL). We then modeled each patient as a day-by-day temporal sequence. Next, the algorithm ‘scanned’ each patient’s entire care sequence to determine the types of treatment cycles followed at each point in time [i.e. CisGem: day 1 and day 8 every 3 weeks (Q3W) versus FOLFOX: day 1 every 2 weeks (Q2W)]. The reconstruction of complete treatment lines was then carried out by ATLAS, based on an analysis of the repetition of cycles over time, their sequence, and their medical relevance.

We then calculated OS and TTNT and estimated the probability of transition between treatment lines as a function of time since inclusion using a multistate model (states being defined as a new treatment line or in-hospital death). For greater visibility, we limited the model to four lines. Patients were then censored at the end of their fourth line to avoid polluting the information on this line.

### Regulatory declaration

This study complied with MR006 (Méthodologie de Référence) and was declared on the Health Data Hub platform (study no. F20221011095840) on 10 November 2022.

### Role of the Funding source

Servier supported the study, but the analysis and interpretation were carried out independently.

## Results

### Incidence and patients’ characteristics

A total of 13 491 were included in the study. Their main characteristics are presented in Table 1. The median age was 72.0 years (IQR 55 to 89 years), including 42.8% of patients aged ≥75 years; 55.9% of patients were men. Concerning comorbidities, 9.4% of patients had cirrhosis, 2.1% viral hepatitis, and 0.7% and 0.5% ulcerative colitis and Crohn’s disease, respectively. One-third of patients (32.1%) had metastases and 20.7% had their first admission before tumor diagnosis via the emergency room. At diagnosis, 11.4% of patients had jaundice, 10.1% ascites, and 4.7% angiocholitis.

**Table 1:** Patients characteristics

Characteristics	Overall population (*N* = 13 491)
Age, mean (IQR)	72 (55 to 89)
Sex: Female, *n* (%)	5943 (44.1)
Admission via emergency department on index stay, *n* (%)	2791 (20.7)
Presence of metastases on index stay, *n* (%)	4329 (32.1)
Symptoms on index stay, *n* (%)	
Jaundice	1535 (11.4)
Ascites	1368 (10.1)
Angiocholitis	634 (4.7)
Nutritional status, *n* (%)	
Severe denutrition	2887 (21.4)
Nutrition intervention	216 (1.6)
Comorbidities, *n* (%)	
Cirrhosis	1270 (9.4)
Other noninfectious nonalcoholic chronic hepatitis	134 (1.0)
HCV	180 (1.3**)**
Hemochromatosis	113 (0.8)
HBV	104 (0.8)
Ulcerative colitis	93 (0.7)
Nonalcoholic steatohepatitis without cirrhosis	80 (0.6)
Crohn’s disease	66 (0.5)
HIV	53 (0.4)
Primary biliary cholangitis	21 (0.2)

HBV, hepatitis B virus; HCV, hepatitis C virus; HIV, human immunodeficiency virus; IQR, interquartile range.

**Figure 2:**
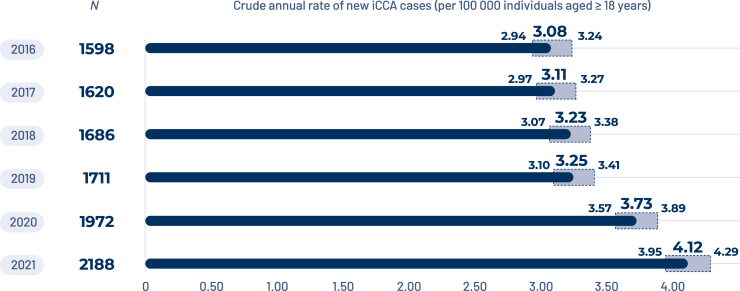
Crude annual rate of new intrahepatic cholangiocarcinoma (iCCA) cases (per 100 000 individuals aged ≥18 years).

The incidence of iCCA continuously increased between 2016 and 2021 from 3.08 per 100 000 individuals aged ≥18 years (95% CI 2.94-3.24) to 4.12 (95% CI 3.95-4.29), reaching 2188 new cases of iCCA in 2021 (Figure 2).

### Outcomes after surgery

In 223 (1.6%) patients who had localized disease and received surgery, the median time to relapse was not reached. The cumulative incidence at 12 months and 24 months after the operation was 21% (95% CI 16% to 27%) and 31% (95% CI 24% to 38%), respectively.

### Type of treatment received

GEMOX (gemcitabine and oxaliplatin) or FOLFOX was the most frequently received L1 chemotherapy (32%) ahead of CisGem (26%). This result fluctuated over the years, with CisGem being less common than GEMOX in 2016 (15% versus 43% of L1, respectively); however, it became the predominant choice from 2020 onward (40% versus 23% of L1, respectively). Regarding L2, oxaliplatin-based doublet (GEMOX or FOLFOX) or FOLFIRI was the most frequently received chemotherapy (38%), ahead of CisGem (16%) and gemcitabine alone (7%).

### Transition between treatment lines

**Figure 3:**
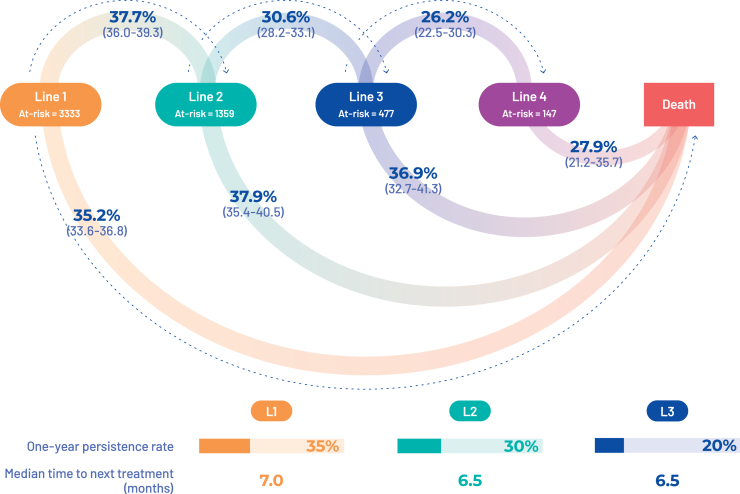
Transition rate between treatment lines.

The analysis was restricted to the 4855 patients with at least one of the following: four chemotherapy sessions, one surgery, and one radioembolization. During the period of analysis, in the 3333 patients receiving L1 treatment, the probability of initiating an L2 was 37.7% (95% CI 36.0% to 39.3%) when the odds of dying were 35.2% (95% CI 33.6% to 36.8%; Figure 3). The 1-year persistence rate in L1 treatment was 35% and the median TTNT was 7.0 months.

When restricted to patients that received CisGem as L1 treatment (*n* = 1116), the 1-year persistence rate in L1 treatment was also 35% and the median TTNT was 7.0 months, and 39.1% (95% CI 36.3% to 42.0%) received L2 treatment.

In the overall population, among 1359 patients receiving L2 treatment, the probability of initiating an L3 was 30.6% (95% CI 28.2% to 33.1%) when the odds of dying were 37.9% (95% CI 35.4% to 40.5%). The 1-year persistence rate in L2 treatment was 30% and the median TTNT was 6.5 months. Among 477 patients receiving third-line treatment, the probability of initiating an L4 was 26.2% (95% CI 22.5% to 30.33%) when the odds of dying were 27.9% (95% CI 21.2% to 35.7%). The 1-year persistence rate in L3 treatment was 20% and the median TTNT was 6.5 months.

In patients receiving treatment, the median OS was 14.0 months from the start of L1, and 10 and 10 months from the start of the second and third lines, respectively.

## Discussion

Our study presents comprehensive real-world data on patients treated for iCCA in France over a 5-year period, allowing us to examine the number of new cases of iCCA, real-world demographic characteristics of patients with iCCA, details of the chemotherapy regimens received, and patient outcomes following these treatments. This update of previously published data confirms the increased incidence of iCCA in France reported in previous studies.^2^^,^^3^ This increase in incidence could be explained both by an increase in certain iCCA risk factors in developed countries, such as chronic liver disease, diabetes, and obesity, and by improvements in diagnostic techniques and, in the case of our analysis of PMSI data, an improvement in the accuracy of diagnostic coding (ICD-10).

Our study confirmed the poor prognosis of patients treated for iCCA with an mOS of slightly >1 year in patients receiving chemotherapy. This is consistent with previous publications based on national or international cohorts, which found mOS of patients treated for advanced iCCA ranging from 7.5 to 13.4 months depending on cohort characteristics.^10^^,^^11^^,^^17^^,^^18^

Although data from PMSI are often limited due to the incompleteness of the data, especially regarding the treatments received, the use of an IA-based algorithm allowed us to generate real-world data on treatment lines in a large number of patients. Thus we were able to generate data on time to line change or death, which is close to the definition of PFS, not only for the first line of chemotherapy but also for subsequent lines, which is very rarely reported in national or international cohorts.^11^ We confirmed the limited activity of CisGem in L1 with a TTNT of 7 months, which is consistent with the ABC-02 trial (8.0 months) and the control arm of the TOPAZ-1 and Keynote-966 trials (mPFS of 5.7 and 5.6 months, respectively).^7^^,^^8^ We also confirmed the low rates of patients who could have access to L2 chemotherapy (37.7%) and subsequent lines and the poor outcomes with these chemotherapies. In addition to providing complementary information to those from clinical trials, these data are useful to study the feasibility of future clinical trials in terms of recruitment in France.

Our study has some limitations, primarily those inherent to studies based on the PMSI, as it is a disease-coding database that can be imprecise. However, a rigorous methodology was applied to limit bias. Additionally, it does not contain biological or anatomopathological data. Furthermore, data from community care are not available, so there is no information on deaths occurring outside the hospital setting, nor are there data on oral therapies. Furthermore, the type of chemotherapy was inferred by ATLAS from the administration schedule (which explains why in L2 GEMOX, FOLFOX, and FOLFIRI, all Q2W, were indistinguishable) but not recorded in the database, so we could not verify this information. Finally, our study was based on data from patients treated before the advent of immune checkpoint inhibitors and broad access to targeted therapy in routine care. Therefore updating data to obtain real-world data on first-line chemoimmunotherapy is an important perspective of our work.

### Conclusion

This large-scale national real-world study confirms the increasing incidence of iCCA in France, the poor prognosis of affected patients, and provides original data on the chemotherapy lines received. It provides interesting data to study the feasibility of clinical trials in France. Updating the data to the period after the emergence of chemoimmunotherapy is an important perspective of this work.
